# Rising prevalence of depression and widening sociodemographic disparities in depressive symptoms among Filipino youth: findings from two large nationwide cross-sectional surveys – CORRIGENDUM

**DOI:** 10.1017/gmh.2025.10019

**Published:** 2025-06-09

**Authors:** Joseph H. Puyat, Divine L. Salvador, Anna C. Tuazon, Sanny D. Afable

Upon publication of Tuazon AC and Afable SD (2025), an error was found in the second sentence of the abstract, which incorrectly reads:

“This study analyzed nationally representative survey data from 2013 (*n* = 10,949) and 2021 (*n* = 19,178) ….”

The correct sentence should be:

“This study analyzed nationally representative survey data from 2013 (*n* = 19,178) and 2021 (*n* = 10,949) ….”

The article has been updated to reflect the correct information.
