# Supplementary material for: Rising prevalence of depression and widening sociodemographic disparities in depressive symptoms among Filipino youth: findings from two large nationwide cross-sectional surveys
Source: Glob Ment Health (Camb). 2025 Apr 14;12:e51. doi: 10.1017/gmh.2025.39 (PMC12116253; doi:10.1017/gmh.2025.39)
Supplement: Puyat et al. supplementary material [file S2054425125000391sup001.docx]

Online Supplement to *Rising Prevalence of Depression and Widening Sociodemographic Disparities in Depressive Symptoms among Filipino Youth: Findings from Two Large Nationwide Cross-Sectional Surveys*

| Item # | CES-D items in the  2013 Young Adult Fertility and  Sexuality Study (YAFS4) | CES-D items in the  2021 Young Adult Fertility and  Sexuality Study (YAFS5) | Rarely or not at all | Sometimes | Often |
| --- | --- | --- | --- | --- | --- |
| 1 | My appetite was poor | Your appetite was poor | 0 | 1 | 2 |
| 2 | I felt depressed | You felt depressed | 0 | 1 | 2 |
| 3 | I felt that everything I did was an effort | You felt that everything you did was an effort | 0 | 1 | 2 |
| 4 | My sleep was restless | Your sleep was restless | 0 | 1 | 2 |
| 5 | I felt happy* | You felt happy* | 0 | 1 | 2 |
| 6 | I felt lonely | You felt lonely | 0 | 1 | 2 |
| 7 | I felt people were unfriendly | You felt people were unfriendly. | 0 | 1 | 2 |
| 8 | I enjoyed life* | You enjoyed life* | 0 | 1 | 2 |
| 9 | I felt sad | You felt sad | 0 | 1 | 2 |
| 10 | I felt that people dislike (do not like) me | You felt that people dislike (do not like) you | 0 | 1 | 2 |
| 11 | I could not get going | You could not get “going” | 0 | 1 | 2 |
| 12 | I felt hopeful about the future* | - | 0 | 1 | 2 |

The above scale items were adopted from the shortened version (Kohout *et al.* 1993) of the Center for Epidemiologic Studies Depression Scale (CES-D) developed by Radloff (1977). The CES-D scale items in YAFS4 and YAFS5 are the same except for differences in the use of lead-pronouns (i.e. “I” and “My” versus “You” and “Your”) and the omission of the 12^th^ item YAFS5. Both scales have yielded good internal consistency reliability as indicated by Cronbach’s alpha of 0.77 (YAFS5) and 0.75 (YAFS4) (Puyat *et al.* 2021).
